# Diversity of *Linum* genetic resources in global genebanks: from agro-morphological characterisation to novel genomic technologies – a review

**DOI:** 10.3389/fnut.2023.1165580

**Published:** 2023-06-01

**Authors:** Vikender Kaur, Mamta Singh, Dhammaprakash Pandhari Wankhede, Kavita Gupta, Sapna Langyan, Jayaraman Aravind, Boopathi Thangavel, Shashank Kumar Yadav, Sanjay Kalia, Kuldeep Singh, Ashok Kumar

**Affiliations:** ^1^Division of Germplasm Evaluation, Indian Council of Agricultural Research-National Bureau of Plant Genetic Resources, New Delhi, India; ^2^Department of Biotechnology, Ministry of Science and Technology, Government of India, New Delhi, India

**Keywords:** characterisation, genetic resources, genebank collections, genomics, linseed, flax, nutrition enrichment

## Abstract

Linseed or flaxseed is a well-recognized nutritional food with nutraceutical properties owing to high omega-3 fatty acid (α-Linolenic acid), dietary fiber, quality protein, and lignan content. Currently, linseed enjoys the status of a ‘superfood’ and its integration in the food chain as a functional food is evolving continuously as seed constituents are associated with lowering the risk of chronic ailments, such as heart diseases, cancer, diabetes, and rheumatoid arthritis. This crop also receives much attention in the handloom and textile sectors as the world’s coolest fabric linen is made up of its stem fibers which are endowed with unique qualities such as luster, tensile strength, density, bio-degradability, and non-hazardous nature. Worldwide, major linseed growing areas are facing erratic rainfall and temperature patterns affecting flax yield, quality, and response to biotic stresses. Amid such changing climatic regimes and associated future threats, diverse linseed genetic resources would be crucial for developing cultivars with a broad genetic base for sustainable production. Furthermore, linseed is grown across the world in varied agro-climatic conditions; therefore it is vital to develop niche-specific cultivars to cater to diverse needs and keep pace with rising demands globally. Linseed genetic diversity conserved in global genebanks in the form of germplasm collection from natural diversity rich areas is expected to harbor genetic variants and thus form crucial resources for breeding tailored crops to specific culinary and industrial uses. Global genebank collections thus potentially play an important role in supporting sustainable agriculture and food security. Currently, approximately 61,000 germplasm accessions of linseed including 1,127 wild accessions are conserved in genebanks/institutes worldwide. This review analyzes the current status of *Linum* genetic resources in global genebanks, evaluation for agro-morphological traits, stress tolerance, and nutritional profiling to promote their effective use for sustainable production and nutrition enhancement in our modern diets.

## Introduction

Linseed or flax (*Linum usitatissimum* L.) has been cultivated for seed oil and stem fiber since ancient times across the world. Globally, six countries (Canada, Kazakhstan, Russia, China, USA, and India) are the major producers (1). At present, flax (fiber-type) ranks as the third largest textile crop, whereas linseed (oil-type) ranks the fifth among the oil crops in the world (1). True to the species name ‘*usitatissimum*’ meaning ‘very useful,’ linseed/flax stem, seeds, and seed oil have a wide range of applications in the preparation of food, nutritional and industrial products. The oil has wide industrial utility in paints and varnishes because of its unique drying properties attributable to its distinctive fatty acid composition (2). Other diverse uses of linseed oil include the manufacturing of hardboards, brake linings, printing ink, linoleum, and soaps. Fiber flax is used in handloom, textiles, and polymeric composites owing to its natural fibers endowed with unique luster and tensile strength (3, 4). The utilization of linseed plants for food, feed, fiber, and value added products has been reviewed comprehensively in the recent past (5, 6).

Linseed has emerged as a well-recognized nutrition-rich food because of its high omega-3 fatty acid (α-linolenic acid; ALA) content, dietary fiber, high quality protein, and lignans. With respect to omega-3 fatty acids, linseed is considered one of the richest plant-based sources and has around 55% ALA of the total fatty acids (7, 8). It also has an impressive omega 6/omega 3 fatty acid ratio of 0.3:1. As the modern-day diet predominates high omega 6 fats, nutritionists recommend a higher intake of essential omega 3 fats in food, which offer tremendous health benefits. Linseed has high fiber content (27.4%) along with high protein content (18.29%) (5). The seeds contain up to 800 times more lignans than other plant foods (9) and are principally composed of secoisolariciresinol diglucoside (SDG) (294–700 mg/100 g) (10–12). ALA together with SDG and dietary fiber has been reported to lower the risk of multiple chronic ailments such as heart diseases, stroke, hormonal disorders, cancer, diabetes, and rheumatoid arthritis (13–16). Currently, cancer is ranked as the second most important cause of death worldwide leading to a heavy global economic toll estimated at $1.16 trillion *per annum*. Over 2 million new cases were diagnosed globally in the year 2020, of which 24.5% accounted for breast cancer (17). Daily consumption of 25 g ground flax or 50 mg SDG has been found to be associated with a decreased risk of breast cancer, its recurrence, and mortality risk among survivors (10, 18). A survey reported increased intake of flaxseed in regular diets in cancer patients (19) since people prefer to use natural therapies in addition to conventional medical treatments for the management of the disease. The integration of linseed into the food chain as a functional food is evolving continuously, and it is popularly being called a ‘nutritional punch’. Whole seeds, milled powder, extracted oil, and mucilage are extensively used as nutritional additives in the preparation of baked/ready-to-eat cereal products, bars, salad dressings, bread, muffins, sweets, processed meat and spaghetti, etc. (5). Renewed interest has led to an increase in consumer demand for linseed-based products not only for culinary use but also for novel industrial applications, such as geotextiles, biopolymers, and biofuels. However, unpredictable environmental stresses, such as drought, salinity, heat, diseases, and pest pressure, result in huge losses in yield and quality of oil/fiber (20–23). Hence, in the near future, climate change may exert strong pressure on flax breeding programs to adapt cultivars to changing conditions at an accelerating rate. Furthermore, the narrow genetic base of modern cultivars poses a major constraint to achieving sustainable yields to cater to diverse needs. Genebank collections of cultivated and wild species of the genus *Linum* from natural diversity-rich areas are important sources of genetic diversity for finding new alleles to meet new environmental challenges. Keeping this in view, the present review article is intended to apprise (1) an overview of the origin, domestication, taxonomy, and gene pool; (2) the status of global *ex-situ* collections of *Linum* sp., (3) trait-specific accessions identified through germplasm evaluation for agro-morphological traits, nutrition profiling, and biotic and abiotic stress tolerance in target environments to promote the effective use of these materials; (4) enhancement of the use of trait specific germplasm through the application of recent advances in genomic resources, molecular and biotechnological tools; and (5) documentation and access to germplasm/information through a global exchange system to facilitate transboundary flow.

## Origin and domestication

Vavilov (24) described two centers of origin based on two distinct morphotypes, linseed (oil) type (short stature, bushy, profusely branched, and small seeded for the seed or oil purpose) originated in south-western Asia comprising India, Afghanistan, and Turkey and the fiber flax type (erect, long, pliable, unbranched stem with few branches restricted to top and bold seeded for fiber purpose) originated in the Mediterranean region including Asia Minor, Egypt, Algeria, Spain, Italy, and Greece. Later, Zeven and de Wet (25) and Damania et al. (26) reported Central Asia as the primary center of origin and the Mediterranean region as the secondary center of origin.

The domestication and subsequent spread of linseed across continents have not been clearly delineated. However, the archaeological evidence suggests that it was domesticated around 8,000 years ago in the Fertile Crescent and consequently spread to Europe and the rest of the world (27). Other dominant regions of diversity include the Indian subcontinent, Abyssinia, and the Mediterranean (24), where the present-day domesticated form, *L. usitatissimum*, originated from the wild ancestor *L. bienne* in geographic isolation. An independent domestication event in Central Asia (Indo-Afghan region) resulted in the development and production of fiber varieties as reported by Duk et al. (28). Crops, such as linseed, having multiple utilities may carry variable domestication signatures. Therefore, multiple domestication events have been suggested to be associated with different regions and included lineages that contain oil, fiber, and winter varieties (29). The long-term domestication of stem fiber and seed oil has diversified it into different types, including fiber, oil, and intermediate (dual-purpose) type (30–33). The first flax varieties were reported to be oil type, and later domestication for fiber use was reported, and multiple paths of flax domestication have been suggested (29, 34, 35). Very recently, Guo et al. (36) also confirmed that oil flax is the ancestor of cultivated flax and revealed signatures of artificial selection during the oil-to-fiber type transition.

## Taxonomy, gene pool, and interspecific hybridization

*Linum* is the largest genus in the family *Linaceae* and consists of nearly 230 species with diverse chromosome numbers ranging from 2n = 16, 18, 30, 36, and 60 or more (37, 38). The scientific names and classifications used for *Linum* taxa in local, national, or regional floras are not consistent. The Global Biodiversity Information Facility lists only 252 accepted species names and 22 doubtful species names in the genus *Linum* (39), whereas Turkish and European floras list more species. The database “Plant List” (40) includes 460 scientific plant names of species rank for the genus *Linum* of which 108 are accepted species names and 229 are unresolved. Many synonyms exist and are used in scientific communications without cross-references. For example, *L. bienne* and *L. angustifolium* are considered synonymous in several databases, such as GRIN, Flora Europaea, The Plant List, and NCBI taxonomy; however, genome analysis based on molecular markers showed that *L. bienne* is a subspecies of *L. usitatissimum* rather than a separate species (41, 42). Several morphological, cytological, and molecular characterizations have revealed that *L. bienne* is the wild progenitor of cultivated flax (35, 43–52).

**Figure 1:**
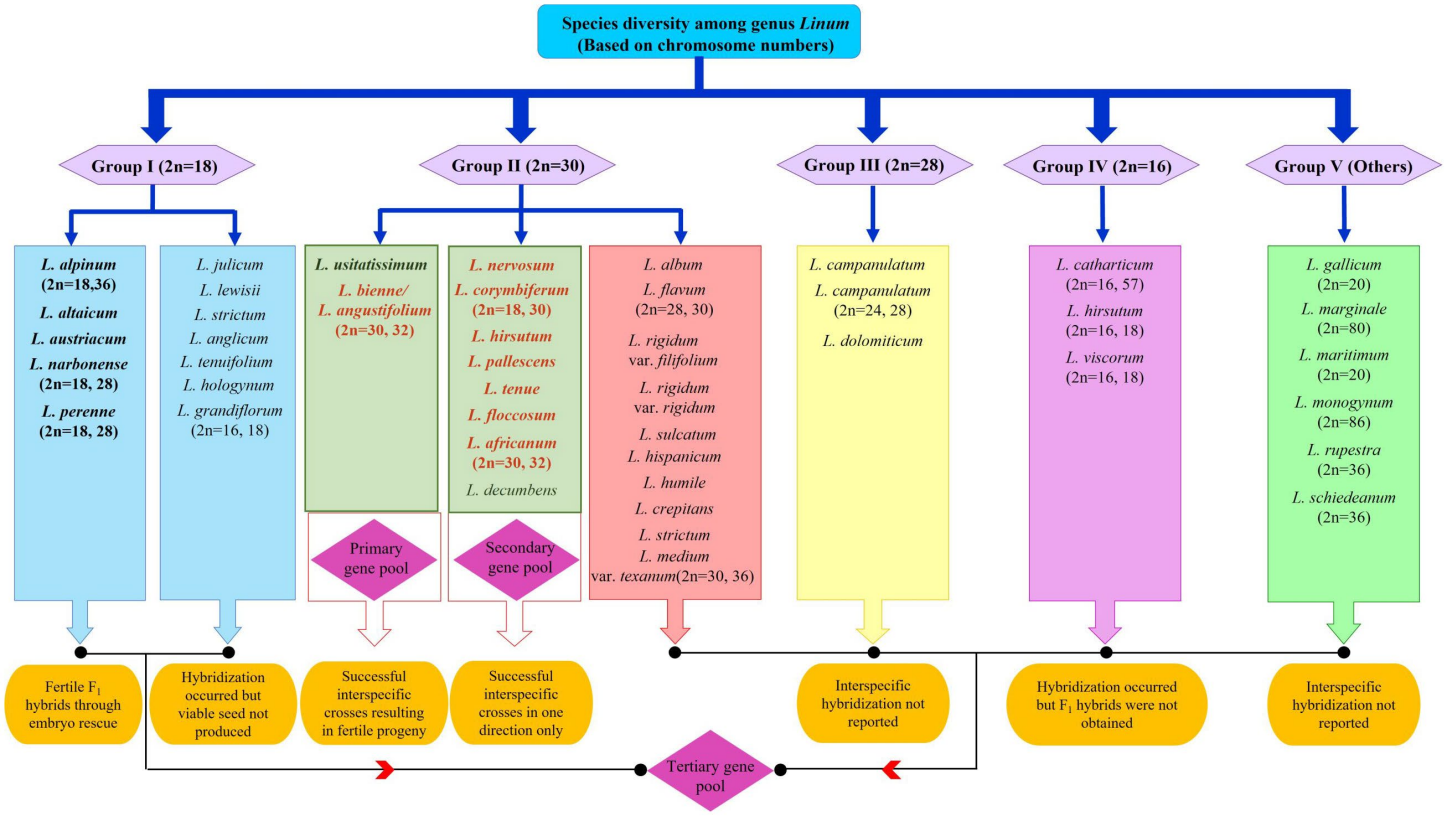
Species diversity in the genus *Linum* and status of interspecific hybridization among different gene pools. Grouping is based on chromosome numbers as proposed by Gill (37). Bold letters indicate the greatest potential to hybridize with cultivated linseed. The colored font indicates most potential wild species for utilization in breeding programs as donors for specific traits of economic importance such as oil and fiber quality, disease resistance, and abiotic stress tolerance.

Despite many taxonomic, cytological, and evolutionary studies conducted for the genus *Linum,* flax genetic resources could not be classified into distinct gene pools as proposed by Harlan and de Wet (53) or modified by Gepts and Papa (54). In addition to classification, there are ambiguities in the identification of species, mainly *L. perenne, L. lewisii*, *L. flavum,* and *L. africanum;* hence, taxonomic revisions had been suggested (55, 56). Jhala et al. (57) also emphasized that species delimitation needs clarification for classification and efficient conservation of genetic diversity in the genus *Linum*. Thus, the absence of a coherent taxonomic review for reference to communicate interspecific diversity has made *Linum* systematics and taxonomy unclear. Understanding this requirement, a recent conspectus was presented by Fu (58) using flax as a case, and different flax species were assigned to various gene pools based on the existing literature. *L. usitatissimum* and *L. bienne* were categorized under the primary gene pool as they are interfertile and share the same chromosome number. Interspecific crosses between *L. usitatissimum* and other species, such as *L. africanum, L. angustifolium, L. corymbiferum, and L. decumbens,* having n = 15 chromosomes have been reported as successful (45). In addition, successful crosses with *L. nervosum*, *L. pallescens*, *L. africanum*, *L. corymbiferum*, *L. decumbens*, *L. hirsutum*, *L. floccosum*, and *L. tenue* have been reported in either direction based on crossability studies of cultivated flax with wild species (38, 59). The tertiary gene pool includes all 200 other species of the genus *Linum* that cannot hybridize with cultivated flax but could be exploited using advanced biotechnological tools (Figure 1). Several *Linum* species have the potential for beneficial trait introgression such as for lowering the linolenic acid content (*L. tenuifolium*, *L. sulcatum, L. hudsonoides*); drought and cold hardiness (*L. perenne*); resistance to linseed bud fly and *Alternaria* blight (*L. grandiflorum, L. bienne*); oil, fiber quality, and yield improvement (*L. bienne*), number of tillers (*L. strictum*); resistance to rust (*L. grandiflorum, L. bienne, L. africanum, L. creptans, L. flocossum, L. gallicum, L. marginale, L. perenne, L. strictum, L. tenue, L. trigyna L. alpinum, L. corymbiferum, L. hispidum*)*; and* medicinal use as purgative (*L. cartharticum* L.) (48, 57, 60–62). The latest interest has been to explore the cut flower potential of perennial flax (*L. austriacum*, *L. perenne*, and *L. lewisii*) for ornamental use in floral arrangements (62). Thus, despite its huge potential, interspecific hybridization in *Linum* is hitherto unexplored to a large extent. A well-defined breeding program aided by biotechnological and molecular biology techniques is required to harness the potential of the wild gene pool and support germplasm enhancement.

## Global *ex situ* holdings of *Linum* genetic resources

**Figure 2:**
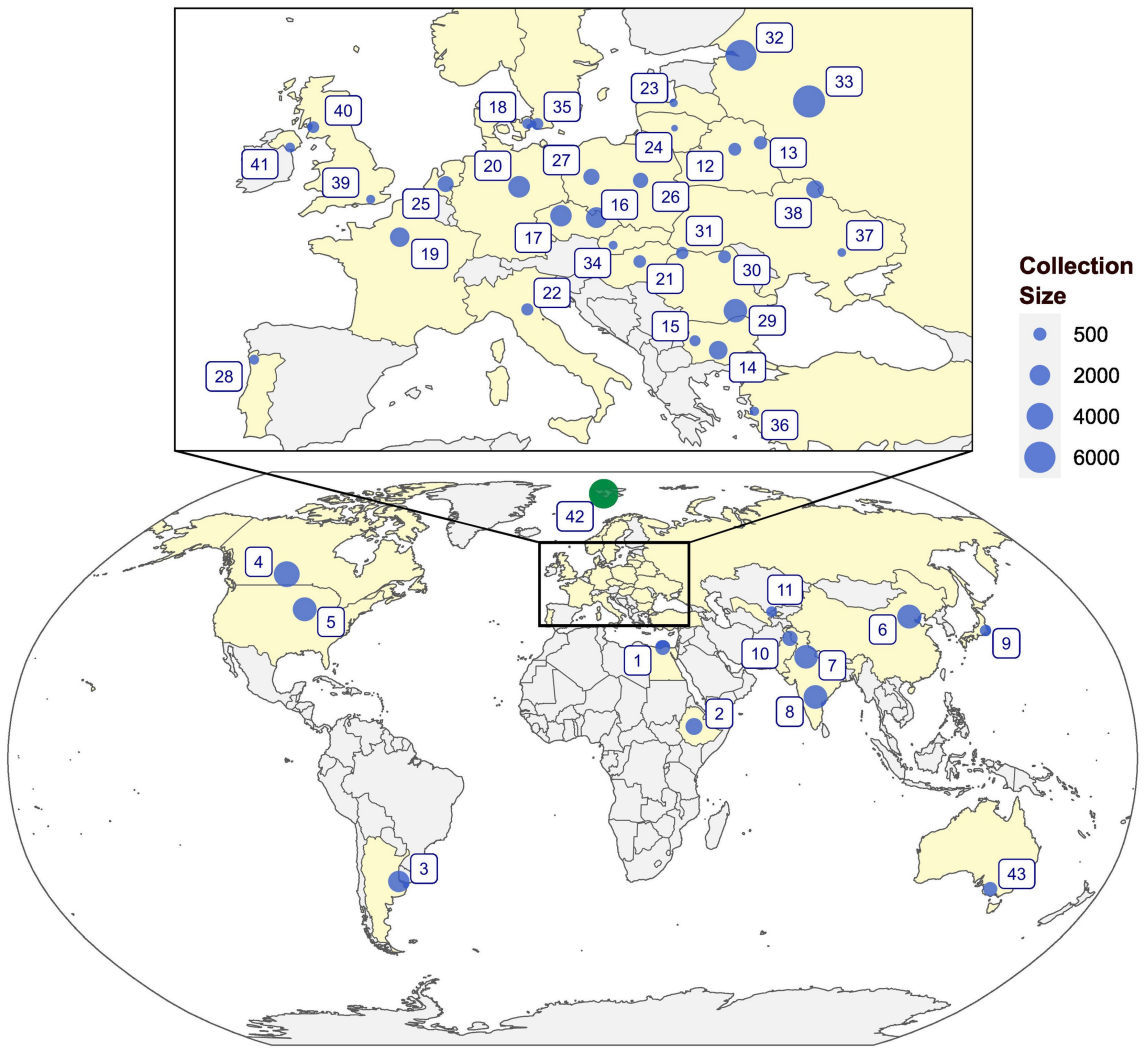
An overview of the global genebanks and institutes holding major collections of *Linum* genetic resources. 1: NGBGR, Egypt; 2: EBI, Ethiopia; 3: BG-CNIA, Argentina; 4: PGRC, Canada; 5: NCRPIS, United States; 6: ICS-CAAS, China; 7: NGB, ICAR-NBPGR, India; 8: AICRP, ICAR-IIOR, India; 9: NARO, Japan; 10: BCI, Pakistan; 11: UzRIPI, Uzbekistan; 12: NA, Belarus; 13: NA, Belarus; 14: IPGR, Bulgaria; 15: ABI, Bulgaria; 16: AGRITEC, Czechia; 17: CRI, Czechia; 18: NA, Denmark; 19: INRAE-VERSAILLES, France; 20: IPK, Germany; 21: NODiK, Hungary; 22: CREA-CI-BO, Italy; 23: LSFRI, Latvia; 24: LIA, Lithuania; 25: CGN, Netherlands; 26: IHAR, Poland; 27: IWNIRZ, Poland; 28: BPGV-INIAV, Portugal; 29: NARDI, Romania; 30: BRGV, Romania; 31: SCDA Livada, Romania; 32: VIR, Russia; 33: VNIIL, Russia; 34: SVKPIEST, Slovakia; 35: NordGen, Sweden; 36: AARI, Turkey; 37: IOK, Ukraine; 38: IBC, Ukraine; 39: RBGK, United Kingdom; 40: SRUC, United Kingdom; 41: NA, United Kingdom; 42: SGSV, Norway; and 43: AGG, Australia.

Earlier reviews on *ex situ* collections were presented by Maggioni et al. (63) who provided the status of *ex situ* germplasm of linseed in Europe and Diederichsen (55) for global collections. The N.I. Vavilov Research Institute for Plant Industry (VIR) in St. Petersburg, Russia, and the All-Russian Flax Research Institute (VNIIL) at Torzhok, constitute the largest collections of approximately 6,000 accessions at each institute (55, 64, 65). The Indian National GeneBank (INGB) at the Indian Council of Agricultural Research-National Bureau of Plant Genetic Resources (ICAR-NBPGR), New Delhi, holds about 2,900 accessions (66). In addition, around 2,942 accessions are being maintained by the All India Coordinated Research Project on Linseed, ICAR-Indian Institute of Oilseed Research, Hyderabad, India (https://aicrp.icar.gov.in/linseed/ (67)). The Plant Gene Resources of Canada (PGRC) holds around 3,551 accessions of cultivated and 152 accessions of 25 wild species assembled from 72 countries. Currently, a total of 59,786 germplasm accessions of *L. usitatissimum* and 1,129 accessions belonging to different wild *Linum* species are conserved worldwide (Figure 2; Supplementary Table 1).

## Evaluation of *Linum* germplasm collections

### Agro-morphological traits

Many diversity assessment studies were conducted in PGRC flax collection and a wide range of variations for important traits, such as the onset of flowering (37–69 days), plant height (17–130 cm), and thousand seed weight (2.8 to 11.5 g), were reported (68–71). You et al. (72) phenotyped the PGRC flax core collection for agronomic, seed quality, fiber, and disease resistance traits (Pasmo, Powdery mildew, and *Fusarium* wilt) in multilocation-year environments and reported significant phenotypic variation in both fiber and oil accessions. Similarly, Worku et al. (73) evaluated Ethiopian collection for agronomic traits and Zhuchenko and Rozhmina (65) evaluated flax landraces for fiber quality and enlisted many accessions with superior trait value. In India, agro-morphological characterization and diversity analyses have shown broad range of phenotypic expression and many trait-specific accessions for early flowering, early maturity, oil content, bold seededness, high-test weight, and ALA were identified (5, 74–79). In pale flax, phenotypic diversity was studied by Diederichsen and Hammer (43) in Canadian and Uysal et al. (80) in Turkish germplasm. A high range of variation in pale flax was recorded for traits, such as plant height (38.4–123.3 cm), no. of days until the start of flowering (97–129), days to maturity (178–207), thousand seed weight (1.10–2.70 g), and seed color. Researchers are exploring advanced technologies, such as genomic tools and marker-assisted breeding, to accelerate the development of high-quality linseed varieties with desirable traits.

### Germplasm evaluation for nutritional and nutraceutical traits

Biochemical characterization and evaluation of linseed for nutritional traits have been performed around the world. Green and Marshall (81) studied 214 accessions of linseed for the content of seed oil, seed weight, and fatty-acid composition and observed that the oil concentration in flaxseed was in the range of 33.3–46.4%, which positively correlated with high seed weight. Earlier Zimmerman and Klosterman (82) also reported a similar oil content in 1,175 linseed germplasm accessions. Variation in seed oil content, as well as the fatty acid composition of cultivated flax germplasm accessions at PGRC grown in western Canada, was studied by Diederichsen and Fu (68), Diederichsen and Raney (69), Diederichsen et al. (71, 83) in 2,934 germplasm accessions and a min (31.4%) to max (45.7%) oil content was found to be contributed by brown seeded linseed (2730) and yellow seeded accessions (84). Various Indian researchers (5, 76, 77, 79, 85) evaluated small subsets of linseed germplasm for qualitative and quantitative traits and reported oil content between 29.4 and 42.6%. For ALA content, a range of 48.08 to 57.58% was reported by Bayrak et al. (86) in Turkish and Romanian genotypes, while a similar variation (48.9 to 59.9%) was recorded in a Polish flax collection by Silska (87). In the Indian germplasm, the ALA content varied from 39.5% in germplasm accession IC564687 to 57.1% in IC564631 (85) and to as low as 33.14% (88). The proportion of ALA in a huge set of PGRC flax collection (2,243 accessions) was reported to be 39.6 to 66.7% with a mean of 52.6% (55).

Linseed also has high protein content which generally varies from 10.5 to 31% (7, 11). Flaxseed meal is a significant by-product (de-oiled) extracted from the processing of flaxseed and is generally rich in protein content of up to 40%. Protein contentmay vary with the environmental, genetic factors, processing techniques and is negatively correlated with oil content. Oomah et al. (89) evaluated 109 accessions to determine the composition of carbohydrates, protein, and oil content. Until recently, sparse information has been reported on germplasm evaluation for protein content in linseed. Similarly, the evaluation of linseed germplasm for SDG content has been performed to a limited extent only. A varied amount of SDG content ranging from 12.9 to 14.3 mg/g (90, 91) and 11.9–25.9 mg/g in whole linseed (92) has been reported. Thus, genotypic variation had a profound effect on the seed SDG content.

Recently, seed coat mucilage content has been recognized as a new character of interest for industrial applications. Large scale evaluation for mucilage content (assessed as Mucilage indicator value; MIV) was carried out by Diederichsen et al. (83), who screened 1,689 accessions from the PGRC collection and identified potential genetic resources. Canadian germplasm had been reported to have higher MIV (22.1 to 343.4 cSt mL g^−1^) than North American cultivars (90.6–246.1 cSt mL g^−1^). The scarcity of information on the chemical composition and functional properties of flax mucilage has limited its use in industries (93). However, recently, linseed mucilage has been recognized as having high technological value as a retardant polymer in pharmaceutical applications as well as in the food industry as a cost-effective natural polymer. Table 1 summarizes the major studies reporting variability for agro-morphological, phenological, fiber, and nutritional traits in flax germplasm and sources of trait-specific superior accessions. The health-related properties of linseed and their rejuvenated importance in animal and human nutrition have stimulated research and breeding programs for novel traits in germplasm collections. The current status of this research area is promising, with several studies identifying linseed varieties with high levels of desirable nutritional and nutraceutical traits. The future of linseed germplasm evaluation for nutritional and nutraceutical traits seems promising with an increasing focus on developing high-quality varieties and functional food products that can provide health benefits beyond basic nutrition.

**Table 1:** Potential accessions identified in linseed and fiber flax germplasm for various agro-morphological and nutritional traits.

Traits	Trait value	No. of accessions studied	Promising accessions* (value)	References
Early flowering	<60 days for 50% flowering	111	IC345409 (55.0); IC345397 (55.0), IC345425 (58.5)	(76)
		50	Shival (41.47)	(94)
			EC704 (47.61), EC41741 (55.47)	(95)
		103	IC397953 (58)	(77)
		220	IC0525939 (57.28), IC0523807 (58.44), IC096539 (59.95)	(79)
		198	59 accessions (<57 days)	(73)
		191	IC0096539 (58), IC0096496 (60)	(5)
		58	IC15888, IC113156, IC11310, IC426932, IC113105 (<60 days)	(96)
Early maturity	<120 days	50	Shival (76.30)	(94)
		191	IC0096539 (102), IC0096496 (97)	(5)
		220	IC0523807 (118.26), IC0525939 (116.76)	(79)
		111	IC268349 (89), IC345425 (92)	(76)
		198	30 accessions (<112 days)	(73)
Number of capsules per plant	>150	220	IC0053278 (267.52), IC0384578 (280.26)	(79)
		103	EC541212	(77)
		191	IC0426935 (168.55)	(5)
Thousand seed weight	>8 g		EC41741 (8.679 g), Ruchi (8.393 g)	(95)
		220	EC0041469 (9.51), EC0041700 (10.93), EC0041720 (9.49 g)	(79)
		191	IC0096490, IC0096488, IC0096489, IC0096543 (>8 g)	(5)
		200	CIli 2,719 (~10 g)	(36)
		1,689	CN98192 (10.87 g)	(97)
		36	Bekoki-14 (8.60 g) (Ethiopian germplasm)	(98)
Plant height	Tall (>110 cm)	3,087	CN101419 (130 cm)	(70)
	Dwarf (<45 cm)	3,087	CN95176 (20 cm)	(70)
		111	IC345425 (41.1 cm)	(76)
Primary branches/plant	>10	50	IC54970 (16.20 cm)	(94)
		36	Acc. 13,507 (11.90 cm), 207,789 (13.80 cm), 208,794 (11 cm), 212,512 (13.50 cm) (Ethiopian germplasm)	(98)
Seeds per capsule	>8	50	IC56363 (8.70)	(94)
		198	195 accessions (>9)	(73)
		191	EC0718845, IC0267682	(5)
		60	Acc. No. 10007, 10,008, 10,061, 10,111, 10,119, 10,162, 10,169, 10,185, 10,192, 10,235, 10,064, 10,256, 10,260 (>10) (Ethiopian germplasm)	(99)
Bold capsules	>50mm^2^ capsule area	220	EC0041700 (52.58 mm^2^)	(79)
		191	IC0094487, IC0096488	(5)
Large seed length, width, and seed area			EC41741, EC704, Ruchi	(95)
		200	Cili 2,719 (PI 523932)	(36)
		191	IC0054949, IC0054954, IC0096490 (15.13 mm^2^ seed area)	(5)
		220	EC0041469 (14.04 mm^2^), EC0041700 (14.63 mm^2^ seed area)	(79)
Seed yield/plant		49	Litwania-9 (Litwanian variety), Evelen (French variety)	(100)
		64	Acc. 243,807, 243,810, 244,809, 231,457, 230,822 (Ethiopian germplasm)	(101)
		81	PGRC/E 10104, 10,120 (Ethiopian germplasm)	(102)
		151	Shweta, Gaurav	(103)
Technical stem height	>35 cm	198	45 accessions (<36.50 cm) (Ethiopian germplasm)	(73)
		7	Ariane (97.4 cm), Viking (93.3 cm) (French cultivars)	(104)
		103	EC5412149 (Taller stalk)	(77)
Cell wall (%)			Upto 80% (recombinant inbred line population derived from CDC Bethune/Macbeth)	(105)
Straw yield/plant	>3 g/plant	7	Giza 5 (3.32 g/plant) and Giza 6 (3.16 g/plant) (Egyptian cultivars)	(104)
		7	INA, Emilin, Rolin, Daniela, Madras, Istru	(106)
		49	Litwania-5 (3.29 g/plant)	(100)
Oil content	>40%	191	IC0096490 (42.9), IC0268345 (42.7)	(5)
		111	IC345425 (41.5), IC345447 (41.4), IC345417 (41.4), IC345423 (41.3)	(76)
		103	IC567363	(77)
			IC564681 (42.6)	(107)
		120	CN18973, CN18979, CN19003, CN19005, CN30861, CN97306, CN97307, CN97308, CN97334, CN97366, CN97396, CN97430, CN97430B (>44)	(108)
		243	Upto 48.5% (recombinant inbred line population derived from CDC Bethune/Macbeth)	(105)
		49	Vaiko (45)	(100)
		36	Acc. No. 208425 (40.05) (Ethiopian germplasm)	(98)
		84	IC564681 (42.6)	(85)
		151	Shubhra (45.09), Laxmi-27 (45.06), Mukta (44.94), Shweta (44.25)	(103)
High α-linolenic acid (ALA)	>57%		NuLin™ 50 (68%) PI523353 (57.61%)	(109)
		50	RLC 92 (58.71), Janki (57.45)	(94)
		198	25 accessions (>57%)	(73)
			IC564631 (57.1%)	(107)
			UGG5-5 (63–66%) (Breeding line)	(110)
		120	CN18979, CN18980, CN18989, CN19004, CN19157, CN19158, CN30861, CN97214, CN97393, CN97406, CN97402, CN97424 (>57%)	(108)
		243	Upto 61.7% (recombinant inbred line population derived from CDC Bethune/Macbeth)	(105)
		84	IC564631 (57.1%)	(85)
			EC541221 (66%)	All India Coordinated Research Project on Linseed^#^, IIOR, Hyderabad, India
Low alpha-linolenic acid (ALA)			Linola™ or Solin™ (∼3%)	(111) (112);
			SP2047 (Solin™ line) (2–4%)	(110)
			<29%	(113)
		84	IC564687 (39.5%)	(85, 107)
			Kiran (33.14%)	(88)
High Oleic acid content		84	IC564627 (32%)	(85)
		198	19 accessions (>19%)	(73)
		120	CN96958, CN96974, CN97083, CN97176, CN97238, CN97312, CN97064	(108)
		64	Acc. No. 13545 (21.4%) (Ethiopian germplasm)	(114)
Protein content	>25%	243	Upto 27% (recombinant inbred line population derived from CDC Bethune/Macbeth)	(105)
Mucilage content	Mucilage indicator value (MIV) >200 cSt mL g^−1^	14	CN19004 (246.06), CN18973 (244.05), CN52732 (222.48)	(83)
		1,689	CN98100 (343.4), CN98254 (342.2)	

CN = Canadian number, PGRC (https://pgrc-rpc.agr.gc.ca/gringlobal/search). IC = Indigenous collection; EC = Exotic collection, Indian National GeneBank (http://www.nbpgr.ernet.in:8080/PGRPortal/(S(lzvbzn55u4enjtfhv3dybl3b))/default.aspx). PI = Passport information, Genesys (https://www.genesys-pgr.org/). Ethiopian germplasm (The Ethiopian Institute of Biodiversity Conservation (IBC/ETH); formerly The Plant Genetic Resources Centre Ethiopia, PGRC/E) (http://www.ebi.gov.et/). VNIIL = Russian Genebank (http://www.vir.nw.ru/; http://vniil.narod.ru/).

* Only the accessions having National id/released cultivars are mentioned.

# https://aicrp.icar.gov.in/linseed/.

### Evaluation for major biotic stresses

Among biotic stresses, wilt caused by *Fusarium oxysporum* f. sp. *lini* has been recognized as the most devastating disease worldwide resulting in 80 to 100% loss in yield (115, 116). Differential diversity in *Fusarium* wilt tolerance in flax with higher resistance in accessions from East Asia, moderate resistance in American accessions, lower than average resistance in accessions from Europe and the Indian subcontinent was reported by Diederichsen et al. (117). Although most domesticated varieties are moderately resistant to *Fusarium* wilt (118, 119), the low genetic diversity of flax varieties and climate change may lead to increased aggressiveness of pathogenic breeds, substantially increasing the risk of disease. Another important fungal pathogen is *Alternaria linicola* Groves and Skolko, which is mostly prevalent in northwestern Europe and causes seedling blight (120), whereas *A. lini* Dey is found predominantly in the Indian subcontinent and causes flower and stem blight. Field evaluations under artificial epiphytotic conditions resulted in the identification of either no or very few lines as resistant (84, 121–124).

Powdery mildew, caused by the fungus *Oidium lini* Skoric, is another important disease of flax, which is reported to cause yield losses between 12–38% in India (125), upto 18% in the United Kingdom (126) and 10–20% in Canada (127). Sources of resistance to powdery mildew have been reported (121, 128, 129) in natural hotspot areas (Table 2). Genes conferring resistance to local *O. lini* isolates have been identified in Indian flax breeding lines (152, 153) and Canadian and European oil cultivars (127). Recently, several powdery mildew resistance QTLs have been identified (154, 155).

**Table 2:** Evaluation of linseed germplasm for tolerance to major biotic and abiotic stresses and sources of promising accessions.

Traits	No. of accessions studied	Traits value	Promising accessions*	References
Biotic stress resistance				
Fusarium wilt		Resistant (up to 5% wilt)	OL 1–3, EC1392, JRF 5, Padmini, JRF-3, RLC-2, RLC 33, R-7	(121)
			Ayogi, EC41656, JLS-9, RLC 46, Nagarkot, Padmini, Rashmi, R-552, Surabhi, Sweta, T-397	(130, 131, 132)
	297	Disease Severity Index (DSI)	25 accessions (DSI = 0)	(133)
Powdery mildew	294	Resistant (0.1 to 10% leaf area affected)	EC41562, EC1465, IC16392, Neelum	(121)
		Resistant (0.1 to 10% leaf area affected)	EC41656, EC322646, Meera	(134)
	150	Highly resistant (0% leaf area affected)-21 accessions	Parvati, Jeevan, R-17, RLC-148, RLC-151	(75, 128)
*Alternaria* blight	250	Resistant (1–10% leaf area infected)	EC22672, EC22704, EC41623	(135)
	200	Resistant	NP-8, NP-48	(84)
			Kiran, Jeevan	(136)
Rust	200		EC384154, EC1497, Baner, Nagarkot, JRF-3, Rashmi	(61)
			Hira, Mukta, Neelum, KL-1 (Surbhi)	(137)
			EC77959, JLS (J)-1, Jawahar 17, Jawahar-17, Garima, Himalini, Padmini, Rashmi, Sheela, Shweta, Meera, Surabhi, Nagarkot, Kiran	(138)
Budfly	288	Resistant (10% bud infestation)	EC1392, EC1424, IC15888, JRF-5	(139)
	60		Neela	(140)
	250		EC22596, EC22672, EC22704, EC22823, EC41528, EC41551, EC41593, EC41715, EC41581, EC41595, EC41623, EC26006, EC41404, EC41580, EC41690	(135)
			IC16382	(141)
Abiotic stress tolerance				
Drought stress	96	Thousand seed weight	CN98566	(97)
	115	Thousand seed weight	CN98712, CN98566	(142)
		High grain yield, Stress tolerance index	CN101595	
		Bundle weight	CN101052, CN101419	
	105	Improved root and shoot traits	CN98193	(143)
	105	Yield, mean productivity, and improved stress indices	CN19004, CN19003	(144)
	41	Superior plant height and root trait stability	CN33393, CN18987	(145)
	41	Total root length	CN98946	(145)
	119	Higher mean productivity and Stress tolerance index (STI)	13 lines from population of KO37 (Iranian breeding line) × SP1066 (Canadian breeding line)	(146)
	120	Total root length (Stability index >3)	CN101348, CN30861, CN18994	(147)
		Total root volume (Stability index >4)	CN101348, CN98056, CN18994	
		Root surface area (Stability index >3)	CN101348, CN30861, CN18994, CN33399	
		Yield under drought stress (>95 gm^−2^)	CN19003, CN19004, CN52732, CN100674	
	200	Germination, early root and shoot growth	VNIIL-180, 1,270, Torzhokij 4, VNIIL-409, Crepitam Tabor, XLB, BELADI Y 6903, CIli 1919 (PI 249989), CIli 2038, CIli 2047, ALSEE, MESSENIAS, OLEIFERA, 62/125–4, CIli 1832, TJK04-72 (PI 649756), TJK04-348 (PI 649760)	(148)
Salt stress tolerance	2	More root length, shoot length, and higher proline and peroxidase activity under salt stress	NL-97	(149)
	10	Germination, seedling growth, and ion content	Sar_I_-85 (Turkish cultivar)	(150)
	5	High antioxidant enzymes peroxidase (POD), superoxide dismutase (SOD), ascorbate peroxidase (APX), and malondialdehyde (MDA) content	Ariane (French) and Sakha-1 (Egyptian)	(151)

CN = Canadian number, PGRC (https://pgrc-rpc.agr.gc.ca/gringlobal/search). IC = Indigenous collection; EC = Exotic collection, Indian National GeneBank, (http://www.nbpgr.ernet.in:8080/PGRPortal/(S(lzvbzn55u4enjtfhv3dybl3b))/default.aspx). PI = Passport information, Genesys (https://www.genesys-pgr.org/). VNIIL = Russian Genebank (http://www.vir.nw.ru/; http://vniil.narod.ru/).

* Only the accessions having National id/released cultivars are mentioned.

In the case of linseed rust, caused by the fungus, *Melompsora lini,* field evaluations to identify sources of resistance, physiologic races, and inheritance of resistance have been reported (137, 138, 156–159) (Table 2). Misra (160) and Singh et al. (116) reported that during screening of wild *Linum* species for rust resistance, the species *L. africanum, L. augustifolium, L. creptans, L. flocossum, L. gallicum, L. marginale, L. perenne, L. strictum, L. tenue,* and *L. trigyna* were found resistant to all the races, while *L. mysorense* and *L. pallecum* were found susceptible.

Among insect pests, defoliators, such as semilooper (*Plusia orichalcea* Fabr), Lucern caterpillar (*Spodoptera exigua* Hubn.), tobacco caterpillar (*Spodoptera litura* Fabr.), and Bihar hairy caterpillar (*Spilarctia obliqua* Walk.), appear as sporadic and region-specific pests with high incidence in some cropping seasons. Bud fly (*Dasyneura lini* Barnes) is a key pest of this crop in Asia, particularly India, Pakistan, and Bangladesh, while it appears as a pest of less economic significance for flaxseed/fiber flax in Europe (161). The linseed incidence of *Dasyneura lini* was first recorded in India by Pruthi and Bhatia (162) and has been reported as the main pest causing yield losses upto 90% in central India (163–165). A few accessions were recorded as resistant (below 10% bud fly infestation score) or moderately resistant germplasm under field conditions in deliberate late planting (139, 165–169) (Table 2). Studies have reported that varietal attributes, such as short flowering periods, flower shape, thin sepals, and higher polyphenol content, render resistance or escape from midges (139, 140, 170, 171). However, limited breeding efforts have been taken to improve this complex polygenic trait.

### Evaluation for major abiotic stresses

Drought, salinity, and heat are the major environmental stressors that negatively affect flowering initiation, plant height, seed yield, straw, and fiber yield in linseed. Heller et al. (172) observed that the fiber formation process in the stem is the most intensive in the vegetative growth period till the end of flowering and therefore weather conditions in this period determine the fiber content and quality. Heller and Byczyńska (173) reported a reduction in fiber yield from 35–50% while screening 51 flax cultivars under water limitation to identify superior yielding lines. In most developing countries, linseed is extensively cultivated in rain-fed or moisture-scarce *utera* conditions and therefore, root system architecture plays an important role in determining water and nutrient acquisition from soil. Linseed has a shallower and less aggressive tap-rooted system compared to other oilseeds such as canola, sunflower, and safflower (174, 175). A deeper and dense root system could extract water more efficiently from deep soil and is thus advantageous, particularly in rainfed production areas (144, 176). Differential performance among flax genotypes under different moisture regimes attributed to variations of their root systems has been reported (97, 177, 178). Linseed is predominantly a rainfed crop in India and therefore Indian genotypes are likely to be more drought tolerant owing to long-time divergent selection under moisture-scarce conditions as also corroborated by several studies (97, 142, 147, 179).

Soil salinization significantly affects the growth and distribution of linseed (151). Only a few studies have reported screening of linseed germplasm against salinity-alkalinity stresses to identify salinity-tolerant lines using germination, biomass and K^+^/Na^+^ ratio (149, 150, 180–183), and the underlying biochemical and physiological mechanisms (151) (Table 2). Yu et al. (184) reported differentially expressed genes (DEGs) and saline-alkaline tolerant miRNAs in flax (Lus-miRNAs) for the first time. Wu et al. (185) identified genes that might enhance salt tolerance by increasing root length and improving membrane injury and ion distribution. Lately, Li et al. (148) screened 200 accessions of flax germplasm for salt tolerance and revealed that stress tolerance indices for germination and early root and shoot traits of the oil flax subpopulation were significantly higher than those of the fiber flax subpopulation. They suggested that oil flax may contain more resistance sites related to abiotic stress, and the screening of resistant germplasm and resistance genes should focus more on oil flax.

Heat stress has a significant impact on the climatic adaptation of linseed, particularly superior quality fiber cultivars as it is a cool-season crop. A rise in temperature during flowering and seed filling may lead to necrosis of the ovules, resulting in a reduced seed set (186). Gusta et al. (187) studied genetic variability among flax cultivars in response to temperature stress and reported negative effects on flowering and seed yield. Cross et al. (188) studied the adverse effects of heat stress (>40°C for 5 days) on reproductive organ functioning and reported a decline in boll formation and seed setting although the composition of the oil was not affected. Fofana et al. (189) reported that warmer and drier environmental conditions can cause a lowering of ALA by 5% owing to thermos-sensitivity of the linked enzyme, fatty acid desaturase (FAD2). The molecular mechanism underlying heat stress tolerance in flax is largely unknown. Recently, Saha et al. (20, 190) attempted to describe the role of heat shock factors (HSFs) and DNA hypomethylation with regard to heat stress adaptation in flax.

## Genomic resources in linseed

### Molecular marker resources in linseed

The PCR-based dominant marker systems, such as RAPD, AFLP, and ISSR, have been employed in flax for linkage mapping and genetic diversity studies since the 1990s until the recent past (99, 191–201). In addition, retrotransposon-based inter-retrotransposon amplified polymorphism (IRAP) markers have been developed in flax and utilized for genetic diversity studies (202). The most preferred PCR-based markers, simple sequence repeats (SSRs) (110, 203), were identified in linseed to the tune of a few hundred until 2011 (50, 51, 204–207). From 2012 onward, there was a substantially higher number of SSRs reported in linseed by different research groups largely due to the advances in next-generation sequencing (NGS) technologies. Cloutier et al. (110) reported 1,164 and 342 SSRs from BAC-end genomic sequences (BESs) and ESTs, respectively. Kale et al. (208) used 454 GS-FLX platform for the sequencing of PCR amplicons and designed 290 SSRs. Furthermore, a reduced representation genome sequencing (RRGS) approach was also used to identify 1,574 SSR loci (209). More recently, 24,375 SSR motifs were identified employing a pseudomolecule-scale genome-wide scan of the flax genome for the development of SSR markers (210). From the identified SSR markers, the polymorphic loci have also been identified in different studies which were used for distinguishing flax and linseed cultivars (206, 207). In terms of functional marker resources, 580 regulatory gene-derived simple sequence repeat (ReG-SSR) markers from transcription factor-coding genes and long non-coding RNAs have been identified (211). In wild species of flax, *Linum bienne*, 44 microsatellite loci have been identified using genome skimming; of which, 16 have been used for genotyping six *L. bienne* populations (212). More recently, a few to hundreds of thousands of single nucleotide polymorphic (SNP) marker loci have been unraveled by an array of techniques such as reduced representation sequencing approaches and whole genome resequencing in linseed accessions (36, 78, 143, 213, 214). Detailed information on the major marker resources available in linseed has been shown ( Supplementary Table 2). These resources, especially SNP markers, have enabled the construction of high-density linkage maps, identification of QTLs, and thereby a better understanding of the genetic architecture of several complex traits in linseed (78, 215–219).

### Genetic diversity in linseed germplasm elucidated by molecular markers

Different marker systems, such as AFLP, RAPD, ISSR, IRAP, SSR, and SNPs, have been employed in linseed specifically for studying the genetic diversity (33, 79, 195, 197, 198, 202, 220–222). The genetic diversity studies with sufficiently large germplasm accessions (>100) have been considered in this review. The genetic diversity of 708 accessions of cultivated flax and 10 wild species was studied using polymorphic IRAP markers (202). The 708 accessions of cultivated flax comprised 143 landraces, 387 varieties, and 178 breeding lines from 36 countries. The robust 141 reproducible data points per accession were obtained from 10 polymorphic IRAP primers with 52% polymorphism and 0.34 Shannon diversity index. The study showed the highest genetic diversity in wild *Linum* species (polymorphism: 100% and Jaccard similarity: 0.57), followed by landraces (58%, 0.63), breeding lines (48%, 0.85), and cultivars (50%, 0.81).

Genetic diversity analysis of 168 linseed accessions predominantly of Indian origin was conducted using 50 SSR loci, which unraveled a total of 337 alleles (74). The mean Shannon’s information index for all three populations was 0.23. Similarly, in the case of Ethiopian linseed landraces, IRAP and ISSR markers showed a comparable level of molecular diversity (PIC, 0.16; GD, 0.19) in 203 accessions (222). The genetic diversity of the PGRC flax core collection was studied using 448 microsatellite markers (33). The genetic structure of the core set showed two major groups with six sub-groups having weak population differentiation, weak relatedness (mean = 0.287), and abundant genetic diversity in the total panel (5.32 alleles per locus). The sub-groups were found to have a high proportion of private alleles. Similarly, 350 globally distributed flax genotypes were studied using 6,200 SNP markers, which clustered the 350 accessions into seven sub-populations with moderate genetic diversity (average H = 0.22 and I = 0.34) (221). There was a significant positive correlation (r = 0.30 and *p* < 0.01) between genetic and geographic distances in the whole collection. Overall, the moderate to low genetic diversity reported in different studies on linseed using varied marker systems are as anticipated and could be accounted for the self-pollinated nature of the crop.

### QTL mapping and genome-wide association analysis

In one of the earliest QTL mappings for *Fusarium* wilt resistance in flax using a doubled-haploid population, two AFLP markers (afB13 and afXR6) were found tightly linked to flax rust resistance (223). A linkage map-based QTL study with 329 SNP and 362 SSR markers using a RILs population of a cross of two Canadian varieties, CDC Bethune and Macbeth, had unraveled 20 QTLs for 14 traits comprising oil, fatty acid, iodine, and protein content as well as fiber quality traits (105). Interestingly, one SSR marker Lu2031 on LG4 (chromosome 4, coordinates: 14489225–14489333) was found to be linked to QTLs of five different traits including cell wall (fiber components), straw weight, seeds per boll, days to maturity, and yield (105, 217). Plant height and technical length are crucial traits for fiber flax. A total of 19 QTLs were identified for both traits following linkage map-based QTL analysis using two RIL populations and a total of 4,497 SNPs were anchored on 15 linkage groups with an average marker density of 2.71 cM (224). A high-density linkage map of flax was constructed using 112 F_2_ plants and 2,339 specific-locus amplified fragment (SLAF) markers on 15 linkage groups with a total length of 1483.25 cM and a mean distance of 0.63 cM between two adjacent markers (216). The study also helped map 12 QTLs for 6 flax fiber-related traits. In biotic stress, three QTLs have been identified for powdery mildew resistance located on LG1, 7, and 9 accounting for 97% of the phenotypic variation and suggesting a dominant gene action (225). Until 2020, 313 QTLs for quantitative traits comprising seed yield & agronomic traits (155 QTLs), seed quality (75 QTLs), fiber (11 QTLs), and diseases (72 QTLs) have been identified in flax, most of which were mapped on chromosome-scale pseudomolecules (217).

In addition to biparental QTL mapping, genome-wide association studies (GWAS) have been extensively employed in flax for the genetic dissection of complex traits. In the early years of last decades, SSR markers have been used for association mapping of seed weight trait in linseed, which identified five SSR markers associated with the trait (50). Later, considerably high numbers of SNPs identified by reduced representation sequencing and whole genome resequencing approaches have been used in GWAS. The GWAS strategy was reported for genetic dissection of seed weight trait in flax by two independent research groups, unraveling the associated SNP markers and important candidate genes such as *PHO1*, *cytochrome P450,* and *ubiquitin-proteosome pathway genes* (36, 214). GWAS was also employed for genetic dissection of flowering time in linseed on 200 accessions of the Canadian flax core collection using 70,935 curated SNPs by single and multi-locus methods (ML-GWAS). A total of 40 quantitative trait nucleotides (QTNs) associated with 27 QTL were identified for flowering time accounting for 3.06–14.71% of trait variation (215). ML-GWAS was also used on a panel of germplasm accessions of the National Genebank of India for dissecting flowering time, maturity, and plant height trait using 68,925 SNPs identified by genotyping by sequencing. The study identified 53, 30, and 27 stable QTNs for flowering time traits, days to maturity, and plant height, respectively (78). GWAS was also successfully employed for identifying genomic regions associated with pasmo resistance (PR) in flax in a panel of 370 core collections. In total, 10 different statistical models identified a total of 692 unique QTNs associated with 500 putative QTLs from six phenotypic PR datasets. Interestingly, 45 of the identified QTLs spanned 85 resistance gene analogs including a large toll interleukin receptor, nucleotide-binding site, and leucine-rich repeat (TNL) type gene cluster (213). In a similar fashion, GWAS have been deployed in the genetic dissection of other complex traits in linseed including fiber-related traits, fatty acid biosynthesis, mucilage and seed hull content, capsule numbers, branch numbers, and other important agronomic traits (21, 214, 226–228). Detailed information on GWAS in linseed has been presented ( Supplementary Table 3). The identified QTLs/QTNs/associated markers/candidate genes are expected to facilitate linseed researchers for the crop improvement and tailoring niche specific, stress tolerant cultivars through genomics/marker-assisted breeding.

### Whole genome sequence

In 2012, a *de novo* assembly of the flax genome sequence of the cultivar CDC Bethune was made available, having an estimated 81% genome coverage from 302 Mb non-redundant sequences (229). However, there was a misassembly observed in several regions at the genome level. This genome assembly was further improved using the BioNano genome (BNG) optical map of CDC Bethune in 2018 (230). The refined scaffold sequences and validation of the BAC-based physical map were performed followed by further scaffolding of BNG contigs to the super BNG contigs. These super BNG contigs were then assigned to the 15 flaxseed chromosomes with the help of the genetic maps. These pseudomolecules constituted total 316 Mb sequences covering 97% of the annotated genes (230). These resources were pivotal in localizing the earlier identified and new QTLs/markers for important traits to the chromosome-scale pseudomolecules (78, 215, 217). More recently, the genome of fiber flax cultivar Atlant was sequenced using the Oxford Nanopore and Illumina platforms reporting the complete assembly with a total length of 361.7 Mb (N50 = 350 kb) and 97.40% completeness (231). Furthermore, a chromosome-scale high-quality genome sequence of another fiber flax cultivar YY5 is reported with HiFi and Hi-C technology, which helped substantially improved the earlier assembly of fiber flax (232). Besides these, scaffold-level genome assemblies of the other three cultivars including linseed ‘Longya-10’, fiber purpose cultivar ‘Heiya-14’, and pale seed color flax are also available (233).

The plastid genome of *L. usitatissimum* was sequenced and assembled showing a typical circular DNA molecule of 156,721 bp length (234). The assembled plastid genome showed a total of 109 unique genes, 2 pseudogenes, 176 SSRs, 20 tandem repeats, and 39 dispersed repeats.

These whole genome sequence assemblies would be valuable resources for further fine mapping of causal genetic variants, precise positioning of markers, and thereby in genomics/marker-assisted breeding in linseed.

## Effective utilization of *Linum* genetic resources and trait information

### *Linum* core collections

The large size of germplasm collections conserved at genebanks poses restrictions for the evaluation, utilization, and maintenance of these resources (235). Therefore, the concept of “core collections” (containing approximately 5–10% of the whole collection) was introduced while preserving the maximum genetic diversity of the entire collection with minimum repetitiveness to promote utilization (236, 237). Diverse germplasm in the form of the core collection has stimulated the research to have more and important insights into the genetic variability conserved in huge collections in genebanks. Approximately 10,000 genetically diverse flax accessions in the form of core collections have been maintained in global genebanks (55). Initially, small collections representing donors for specific quality traits and disease resistance were assembled by Kutuzova (238) and Brutch (64) at The N.I. Vavilov Institute (St. Petersburg). Later van Soest and Bas (239) developed a core set of 84 accessions from 506 fiber flax accessions at The Centre for Genetic Resources at Wageningen in the Netherlands. Diederichsen et al. (70) assembled a PGRC flax core collection comprising 407 accessions encompassing both the oil and fiber morphotypes from a world collection of around 3,500 accessions. This core set has been characterized phenotypically for agronomic, fiber quality, and disease resistance traits (72) and to identify the candidate genes and QTLs for seed quality traits and drought tolerance (33, 50, 142, 147), thus contributing to the efficient utilization of genetic resources.

### Documentation and access to germplasm and trait information

Documentation of data on germplasm accessions and their characterization and evaluation is of pivotal importance to facilitate the sustainable use of genetic resources. World Information and Early Warning System (WIEWS) maintained by the Plant Genetic Resources of Food and Agriculture Organization, Rome, Italy hosts information on major flax collections.^1^ Based on the recommendations by IPGRI (240), most of the genebanks have developed their own documentation systems, wherein the passport data are linked to germplasm accessions. These data are often accessible to the users through genebank databases/websites ( Supplementary Table 1). Figshare repository (Federal Research Center for Bast Fiber Crops, Russia) holds data on quantitative phenotypes of flax.^2^ PGRC has provided full access to the passport, characterization, and evaluation of data of the whole flax collection along with photographs of each accession of the core set.^3^ For European flax collections, the passport data is available in International Flax Database.^4^ The PGR information of Indian linseed collection has been duly documented by ICAR-NBPGR in user-friendly databases such as PGR Portal and mobile/desktop apps Genebank, PGRMap (http://www.nbpgr.ernet.in/PGR_Databases.aspx). The evaluated germplasm and trait-specific accessions have been published through catalogs, workshop proceedings, and notification of germplasm registration (241–246). The Inventory of elite germplasm/genetic stocks is available at the NBPGR website.^5^ The catalogs listing 50 linseed accessions as donors for specific traits including disease resistance (238) and fiber characterization data of 250 flax accessions (64) were published by the Vavilov Institute. The comprehensive dataset of flax accessions from the Russian Federal Research Center for Bast Fiber has been recently published by Rozhmina et al. (247). FIBexDB is a database based on transcriptomic data for flax where the expression pattern for genes and co-expression network are available and pairwise comparison can be made (248).

### Exchange of germplasm and information

**Figure 3:**
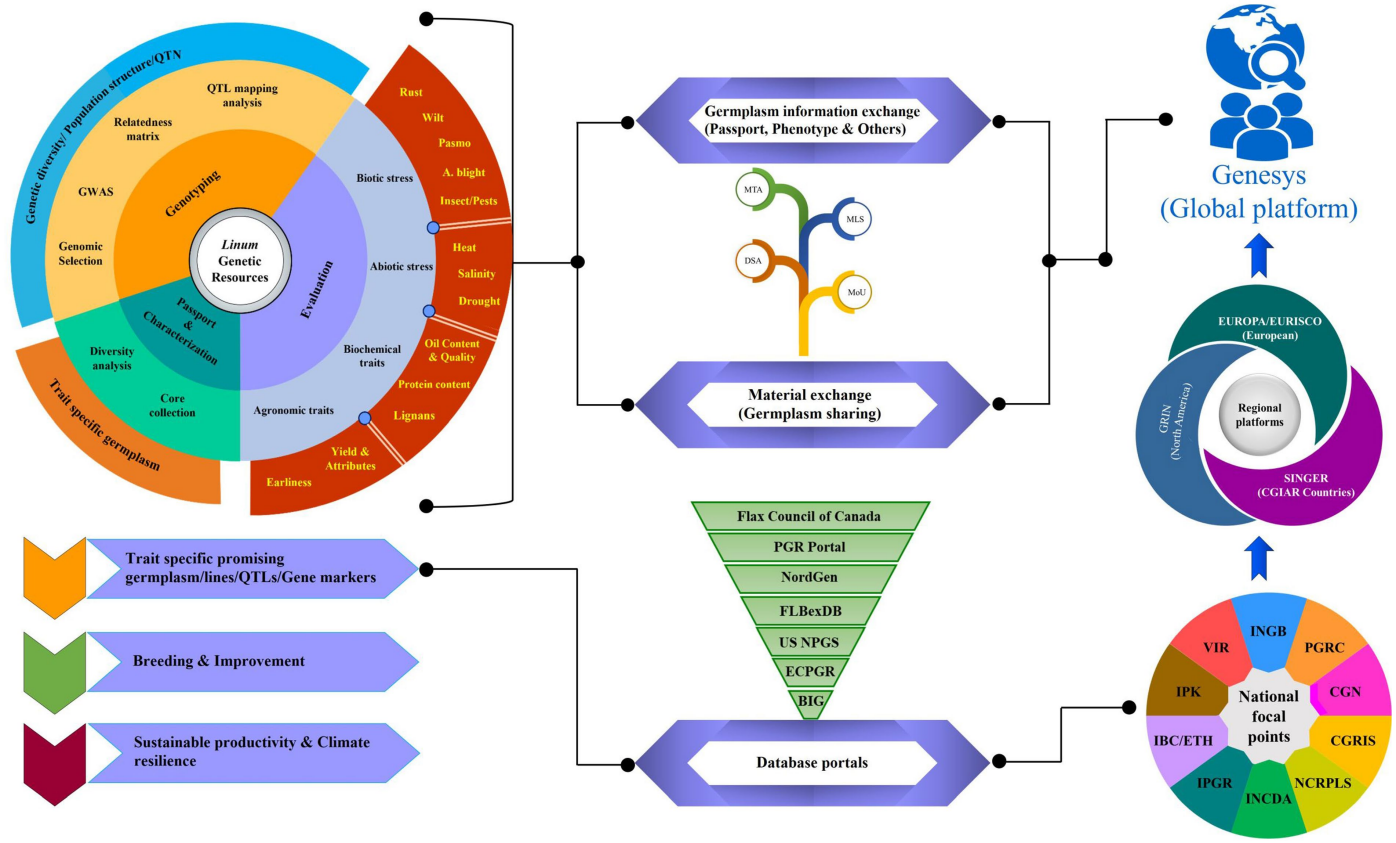
A comprehensive overview of the global network for evaluation, conservation, and exchange of *Linum* genetic resources. The requisite information on conserved genetic resources in genebanks at the national, regional, and global levels is accessible at the respective focal point. Some of the genebanks have been transitioned and progressed from individual institute-level limited operations to multi-institutional, regional, as well as global platforms for access to genetic resources and information. CGIAR, Consultative Group on International Agricultural Research; SINGER, System-wide Information Network for Genetic Resources; EURISCO, European Plant Genetic Resources Search Catalogue; GRIN, Germplasm Resources Information Network; ECPGR, European Central Crop Databases; BIG, Germany’s Federal Information System on Genetic Resources; US NPGS, US National Plant Germplasm System; NordGen, Nordic Genetic Resources Centre; FIBexDB, Plant Fiber Expression Database; INGB-Indian National Genebank; CGN, Centre for Genetic Resources; the Netherlands; VIR, N.I. Vavilov Research Institute of Plant Industry; INCDA, Research Institute for Cereals and Industrial Crops; IBC/ETH, The Ethiopian Institute of Biodiversity Conservation; IPK, The Leibniz Institute of Plant Genetics and Crop Plant Research; Gatersleben; Germany; NCRPLS, North Central Regional Plant Introduction Station; USA; CGRIS, Institute of Crop Germplasm Resources; China; PGRC, Plant Gene Resources of Canada; Saskatoon; MLS, Multilateral System; SMTA, Standard Material Transfer Agreement; MoU, Memorandum of Understanding; DSA, Data Sharing Agreement.

Universal accessibility to information through unified record maintenance in the system is essential to promote the wider use of genetic resources in breeding programs. With the aid of modern information technology tools, a well-developed global network system through inter-operatable data sets is in place now to facilitate the exchange of information and trait discovery. An overview of the evaluation of *Linum* genebank collections through comprehensive phenomics and genomics for the identification of germplasm traits relevant to climate resilience and sustainability is presented in Figure 3. Global genebank systems and databases facilitate the exchange of germplasm and the flow of information for enhanced utilization. Institutional, regional, and global platforms provide inter- operatable data sets and create customized data analysis tools to address the needs of end users and different stake holders (e.g., genebank managers, curators, researchers, breeders, students, and farmers). Accession-level information on plant genetic resources secured in genebanks can be retrieved through FAO WIEWS.^6^ To ease the search of accession level information, a transition from individual genebank website access to multi-institutional/regional portals, such as the European Plant Genetic Resources Search catalogue (EURISCO; http://eurisco.ecpgr.org), the CGIAR system Wide Information Network for Genetic Resources (SINGER; http://www.singer.cgiar.org/), and the Germplasm Resources Information Network (GRIN; http://www.ars-grin.gov/) of United states Department of Agriculture (USDA), has helped a lot. On a global scale, GENESYS (https://www.genesys-pgr.org/) portal integrates data from the website as a single data entry point for users to mine genetic diversity and order germplasm of interest. Currently, it contains information on around 50% of the global accessions; of which, around 24,555 accessions belong to *Linum* sp., and this information is continuously evolving over time (249).

### Conclusion and future perspectives

Germplasm evaluation is a critical aspect of linseed research, as it involves the identification and characterization of genetic resources for the improvement of the crop. Research in linseed germplasm evaluation has been focused on the identification and characterization of diverse germplasm collections for traits, such as oil content, fatty acid composition, seed size, and resistance to biotic and abiotic stresses. This has been accomplished through the use of molecular markers, biochemical analysis, and field evaluations. The present work attempts to review and systematize the existing scientific knowledge about *Linum* genetic resources with respect to the present status of the collection, evaluation, and utilization of major traits of economic importance. The conservation and systematic evaluation of *Linum* genetic diversity is essential to overcome environmental challenges and produce resilient cultivars. Amid the alarming rate of climate change, potential genetic resources from diversity rich and environmentally adapted areas need to be identified for sustainable production. Many studies have reported value-rich and trait-specific promising linseed accession from India, especially high salt and drought tolerant genotypes (68, 142, 147, 148, 179). Therefore, the identification of promising donors for economically important traits and novel molecular tags/genes for important traits in the Indian material will add new information to the domain of knowledge, as of now, only PGRC flax core germplasm has been extensively evaluated for various traits. Moreover, several reports have indicated a narrow genetic base in Canadian linseed cultivars (196, 197, 204), which is an impediment to further breeding progress. In addition, the scarce availability of compatible wild species to incorporate novel variation and the limited molecular breeding have hampered flax yield and quality improvements, thus limiting the competitiveness of the species. Owing to these facts, conventional breeding could result in very low and slow yield gains in linseed. Hence, the utilization of advanced genomic tools to identify key genes and pathways associated with important traits can facilitate the development of molecular breeding approaches for targeted futuristic improvement. Furthermore, the integration of omics technologies in future research and breeding should be emphasized to ensure sustainability in yield and climate resilience. In view of these facts, a mega initiative ‘Leveraging genetic resources of linseed through comprehensive phenotyping and genotyping approaches’ under the Mission program on ‘Minor oilseed of Indian origin’ is being taken up presently in India with financial support from the Department of Biotechnology, Govt. of India, wherein whole INGB linseed collection is being phenotyped and genotyped extensively for key agronomic, quality traits, and major biotic and abiotic stresses. The access to evaluation data, genome-wide availability of molecular markers, and QTLs/QTNs/SNPs/candidate genes underlying important traits will ensure further genetic gains in linseed through markers/genomics-assisted and/or haplotype-based breeding.

## Author contributions

VK, AK, SK and KS conceptualized the theme. VK wrote the initial draft and edited the manuscript. MS, DW, KG, SL, BT, and JA helped in the preparation of manuscript in the respective area of expertise. SY and JA helped in the preparation of figures and tables and editing of the manuscript. AK, KS and VK reviewed the manuscript. All authors contributed to the article and approved the submitted version.

## Funding

This work was supported by funding for the project (No. BT/Ag/Network/Linseed/2019–20) from Department of Biotechnology (DBT), Government of India.

## Conflict of interest

The authors declare that the research was conducted in the absence of any commercial or financial relationships that could be construed as a potential conflict of interest.

## Publisher’s note

All claims expressed in this article are solely those of the authors and do not necessarily represent those of their affiliated organizations, or those of the publisher, the editors and the reviewers. Any product that may be evaluated in this article, or claim that may be made by its manufacturer, is not guaranteed or endorsed by the publisher.

## Footnotes

^1^ http://apps3.fao.org/wiews/

^2^ https://figshare.com/s/86a68ecfacf6872ef239

^3^ http://pgrc3.agr.gc.ca/

^4^ http://www.ecpgr.cgiar.org/database/crops/flax

^5^ http://www.nbpgr.ernet.in:8080/registration/InventoryofGermplasm.aspx

^6^ https://www.fao.org/wiews/en/
