# Supplementary material for: Diversity of Linum genetic resources in global genebanks: from agro-morphological characterisation to novel genomic technologies – a review
Source: Front Nutr. 2023 Jun 1;10:1165580. doi: 10.3389/fnut.2023.1165580 (PMC10267467; doi:10.3389/fnut.2023.1165580)
Supplement: Supplementary file 1 [file Table_1.docx]

Supplementary Material

**Diversity of *Linum* Genetic Resources in Global Genebanks: From Agro-morphological Characterisation to Novel Genomic Technologies – A Review**

Vikender Kaur^1^*, Mamta Singh^1^, Dhammaprakash Pandhari Wankhede^1^, Kavita Gupta^1^, Sapna Langyan^1^, J. Aravind^1^, Boopathi Thangavel^1,2#^, Shashank Kumar Yadav^1^, Sanjay Kalia^3^, Kuldeep Singh^1,4†^ and Ashok Kumar^1^

^1^Indian Council of Agricultural Research- National Bureau of Plant Genetic Resources, New Delhi-110012, India.

^2#^Present address: Indian Council of Agricultural Research- Indian Institute of Oilseeds Research, Rajendranagar, Hyderabad, Telangana-500030, India.

^3^Department of Biotechnology, Ministry of Science and Technology, Government of India, New Delhi-110003, India.

^4†^Present address: International Crops Research Institute for the Semi-Arid Tropics (ICRISAT), Patancheru, Hyderabad, Telangana-502324, India.

*Correspondence: [vikender.kaur@icar.gov.in](mailto:vikender.kaur@icar.gov.in)

**Supplementary Table 1: Global status of conservation of *Linum* genetic resources**

| **S. No.** | **Country** | **Genebank/Institutions** | **No. of accessions of cultivated germplasm** | **No. of accessions of wild *Linum* sp.**  **(no. of sp.)** | **Characterization and evaluation status** | **Website/Reference^$^** |
| --- | --- | --- | --- | --- | --- | --- |
| 1. | Egypt | National Genebank and Genetic Resources of Egypt (NGBGR), Giza | 733 | 0 |  | http://ngb.gov.eg/ |
| 2. | Ethiopia | Ethiopian Biodiversity Institute (EBI), Addis Ababa  (formerly The Plant Genetic Resources Centre Ethiopia, PGRC/E), Ethiopia | 1123 | 0 |  | https://ebi.gov.et/ |
| 3. | Argentina | Banco de Germoplasma, Centro Nacional de Investigaciones Agropecuarias, Instituto Nacional de TecnologÃ­a Agropecuaria, Buenos Aires  (BG-CNIA) | 2226 | 0 |  | https://inta.gob.ar/unidades/211000/banco-base-de-germoplasma |
| 4. | Canada | Plant Gene Resources of Canada (PGRC), Saskatoon | 3551 | 152 (25) | 2759 Intermediate convar. *usitatissimum*, 148 Fiber flax convar. *elongatum*, 65 large seeded convar. *mediterraneum*, 9 dehiscent flax convar. *crepitans*, 127 not categorized. Evaluation done for agronomic, biotic stress and fiber traits (data available on website). | http://pgrc3.agr.gc.ca/ |
| 5. | USA | North Central Regional Plant Introduction Station (NCRPIS), USDA-ARS, Ames, Iowa | 2834 | 164 (36) |  | https://www.cals.iastate.edu/ncrpis |
| 6. | China | National Crop Germplasm Preservation Center, Center for Crop Germplasm Resources, Institute of Crop Science, Chinese Academy of Agricultural Sciences (ICS-CAAS), Beijing | 3003 | 0 |  | <https://www.cgris.net/icgr/icgr_english.html>;  <https://ics.caas.cn/en/facility/nkf/131115.htm>;  <https://ics.caas.cn/zwzzzyzxzz/index.htm> |
| 7. | India | Indian National GeneBank (INGB), ICAR-National Bureau of Plant Genetic Resources (ICAR-NBPGR), New Delhi | 2900 | 10 (3)* | ^#^89% Linseed, 11% Fiber/dual purpose; DUS characterization and preliminary evaluation completed.  Evaluation of whole collection for agronomic, major biotic and abiotic stresses underway. | http://www.nbpgr.ernet.in/  Kaur et al., 2017; Nizar and Mulani, 2015 |
| 8. | India | All India Coordinated Research Project on Linseed (AICRP), ICAR-Indian Institute of Oilseed Research, Hyderabad | 2942 | 2 (1) | 2697 Oil, 214 Dual purpose, 31 Fiber flax.  Characterized for 8 quantitative and 12 qualitative traits. | https://aicrp.icar.gov.in/linseed/ |
| 9. | Japan | Genebank, National Agriculture and Food Research Organization (NARO), Tsukuba-shi, Ibaraki-ken | 294 | 0 |  | http://ss.abr.affrc.go.jp/ |
| 10. | Pakistan | Bio-resources Conservation Institute (BCI), Islamabad | 819 | 0 |  |  |
| 11. | Uzbekistan | Uzbek Research Institute of Plant Industry (UzRIPI), Tashkent | 270 | 0 |  |  |
| 12 | Belarus | Republican Unitary Enterprise 'Scientific Practical Centre of the National Academy of Sciences of Belarus for Arable Farming', Zhodino | 466 | 1 |  | http://www.izis.basnet.by |
| 13 | Belarus | Republican Unitary Enterprise 'The Institute of Flax', Uste | 536 | 0 |  |  |
| 14 | Bulgaria | Institute for Plant Genetic Resources. "K. Malkov" (IPGR), Sadovo, Plovdiv | 1461 | 19 (11) | 23% Linseed, 29% Flax and 46% intermediate type. | http://ipgrbg.com/en/ |
| 15 | Bulgaria | Agrobio Institute (ABI), National Center for Agricultural Science, Kostinbrod | 281 | 2 | 31% Flax, 35% Linseed, Intermediate 32%, Other types 2%. | https://abi.bg/en/ |
| 16 | Czechia | AGRITEC, Research, Breeding and Services, Ltd., Sumperk | 2054 | 0 | 53% Flax, 39% Linseed, 8% Intermediate.  Data on PGR documentation in National documentation system EVIGEZ for passport descriptors and agro-morphological traits. | <https://www.agritec.cz/o-nas>  Maggioni et al., 2002; Pavelek, 2002 |
| 17 | Czechia | Gene bank, Crop Research Institute (CRI), Prague | 2238 | 1 |  | https://www.vurv.cz/ |
| 18 | Denmark | Section for Crop Sciences, University of Copenhagen, Copenhagen | 250 | 0 |  | <https://plen.ku.dk/>  Zhuchenko and Rozhmina, 2000; Diederichsen, 2007 |
| 19 | France | National Research Institute for Agriculture, Food and Environment (INRAE-VERSAILLES) | 1700 | 0 |  |  |
| 20 | Germany | The Leibniz Institute of Plant Genetics and Crop Plant Research (IPK), Gatersleben | 2212 | 93 (26) | 49% Intermediate, 27% Fiber, 7% Linseed, 17% others | http://www.ipk-gatersleben.de/ |
| 21 | Hungary | Centre for Plant Diversity (NODiK), Tápiószele | 409 | 39 (10) |  | http://www.nodik.org/english/ |
| 22 | Italy | CREA-Centro di Ricerca Cerealicoltura e Colture Industriali - Sede di Bologna, Bologna (CREA-CI-BO) | 395 | 0 |  | https://www.crea.gov.it/web/cerealicoltura-e-colture-industriali |
| 23 | Latvia | Latvian State Forest Research Institute 'Silava' (LSFRI), Salaspils | 122 | 0 |  | http://www.silava.lv |
| 24 | Lithuania | Lithuanian Research Centre for Agriculture and Forestry (LIA), Kedainiai | 101 | 0 |  | https://www.lammc.lt/ |
| 25 | Netherlands | Centre for Genetic Resources, The Netherlands (CGN), Wageningen | 946 | 5 (3) | Fiber flax-502, Linseed-450,  Intermediate-7  Database management system Genetic resources Information System of CGN (GENIS) holds Passport and agro-morphological descriptors data | https://www.wur.nl/en/research-results/statutory-research-tasks/centre-for-genetic-resources-the-netherlands-1/plant-genetic-resources/genebank.htm |
| 26 | Poland | Plant Breeding and Acclimatization Institute (IHAR), Blonie | 803 | 14 (6) |  | http://www.ihar.edu.pl/ |
| 27 | Poland | Institute of Natural Fibers and Medicinal Plants (IWNIRZ), Poznan | 979 | 0 | 300-Fiber, 200-Linseed, 31-Intermediate, 200-Unknown Types  Evaluation done for agro-morphological, fiber content, disease (*Fusarium* sp.) resistance | https://iwnirz.pl/ |
| 28 | Portugal | Banco Português de Germoplasma Vegetal (BPGV-INIAV) | 203 | 0 |  | https://www.iniav.pt/bpgv |
| 29 | Romania | National Agricultural Research and Development Institute (NARDI), Fundulea | 2813 | 67 (19) | 2161-Linseed, 1068-Fiber, 71-Intermediate, 545-Unknown  Evaluation for wilt (*Fusarium*) resistance, fiber traits, yield and lodging resistance | https://www.incda-fundulea.ro/ |
| 30 | Romania | "Mihai Cristea" Plant Genetic Resources Bank (BRGV), Suceava | 463 | 2 (2) |  | https://svgenebank.ro/ |
| 31 | Romania | Livada Agricultural Research Station (SCDA Livada) | 420 | 0 |  | https://www.agrim.ro/scda-livada/ |
| 32 | Russia | N. I. Vavilov All-Russian Institute of Plant Genetic Resources (VIR), St. Petersburg | 5765 | 84 (5) | 37% Fiber, 34% Intermediate,  28% Oil/Linseed  Evaluation catalogues published. | <http://www.vir.nw.ru/>  Brutch, 2002 |
| 33 | Russia | All-Russian Flax Research Institute (VNIIL), Torzhok | 6302 | 106 (22) |  | Zhuchenko and Rozhmina, 2000; Diederichsen, 2007 |
| 34 | Slovakia | National Agricultural and Food Centre (NPPC), Research Institute of Plant Production (RIPP), Piestany | 145 | 0 |  | http://www.nppc.sk/index.php/en/ |
| 35 | Sweden | Nordic Genetic Resource Center (NordGen), Alnarp, Sweden | 346 | 0 |  | https://www.nordgen.org/en/ |
| 36 | Turkey | Plant Genetic Resources Department, Aegean Agricultural Research Institute (AARI), Izmir | 157 | 0 |  | https://arastirma.tarimorman.gov.tr/etae/Sayfalar/EN/Anasayfa.aspx |
| 37 | Ukraine | Institute of Oil Crops, The Ukrainian Academy of Agrarian Sciences (IOK), Zaporizka oblast | 105 | 35 (5) |  | http://imk.zp.ua/ |
| 38 | Ukraine | Institute of Bast Crops (IBC), The Ukrainian Academy of Agrarian Sciences, Sumska oblast, Hlukhiv | 1301 | 22 (10) |  | http://ibc-naas.com/ |
| 39 | United Kingdom | Millennium Seed Bank - Royal Botanic Gardens, Kew (RBGK) | 2 | 153 (50) |  | https://www.kew.org |
| 40 | United Kingdom | Department of Plant Sciences, Scotlands Rural College (SRUC), Auchincruive-Ayr | 350 | 0 |  | https://www.sruc.ac.uk/courses-training/campuses/sruc-auchincruive-ayr/ |
| 41 | United Kingdom | Northern Ireland Horticultural and Plant Breeding Station, Loughgall, Armagh | 200 | 0 |  | - |
| 42 | Norway | Svalbard Global Seed Vault (SGSV), Longyearbyen, Svalbard | 4927 | 158 (32) | Safety duplicates | https://seedvault.nordgen.org |
| 43 | Australia | Australian Grains Genebank (AGG), Agriculture Victoria, Horsham, Victoria | 639 | 0 |  | https://agriculture.vic.gov.au/crops-and-horticulture/the-australian-grains-genebank/about-the-australian-grains-genebank |
| **Total** | |  | **59786** | **1129 (267)** |  |  |

^$^Source database are FAO WIEWS and GENESYS except for NBPGR (from PGR portal) and ABI, INRAE-VERSAILLES, SCDA Livada (from ECPGR, 2001).

*Seed multiplication for augmentation of *L. bienne* (12 accessions), *L. marginale* (2 accessions), *L. grandiflorum* (1 accession), *L. altaicum* (1 accession), *L. maritimum* (1 accession) and *L. corymbiferum* (1 accession) is under progress.

^#^M. Singh and V. Kaur, INGB, ICAR-NBPGR, India, personal communication, December, 2022.

**Supplementary Table 2: Markers resources in linseed**

| **Marker resources** | **Species** | **Method used** | **Number** | **Reference** |
| --- | --- | --- | --- | --- |
| Inter-retrotransposon  amplified polymorphism (IRAP) | *Linum usitatissimum* and 10 wild *Linum* species. | Long Terminal  Repeat (LTR) retrotransposon cloning and sequencing | 76 | Smykal et al., 2011 |
| Genomic SSRs | *Linum usitatissimum* | BAC-end sequences, BESs | 1,164 | Cloutier et al, 2012 |
| EST SSRs | *Linum usitatissimum* | ESTs sequences | 342 | Cloutier et al, 2012 |
| Genomic SSR | *Linum usitatissimum* | Sequencing of PCR amplicons by 454 GS-FLX platform | 290 | Kale et al., (2012) |
| Genomic SSR | *Linum usitatissimum* | Reduced representation genome sequencing | 1574 | Wu et al., 2017 |
| Genomic SSR | *Linum usitatissimum* | Scanning of pseudomolecule-scale genome assembly | 24,375 | Pan et al., 2020 |
| Regulatory gene-derived SSR (ReG-SSR) | *Linum usitatissimum* | Scanning of transcription factor-coding genes, and long non-coding RNAs | 580 | Saha et al., 2019 |
| Plastid SSRs | *Linum usitatissimum* | Plastid genome sequencing and assembly | 176 | de Santana Lopes et al., 2018 |
| Genomic SSR | *Linum bienne* | Genome skimming | 44 | Landoni et al., 2020 |
| SNPs | *Linum usitatissimum and*  *L. bienne* | 454 pyrosequencing and genomic reduction | 1067 | Fu and Peterson, 2012 |
| SNPs | *Linum usitatissimum* | Genotyping-by-sequencing of 8 flax genotypes | 55,465 | Kumar et al., 2012 |
| SNPs | *Linum usitatissimum* | specific-locus amplified fragment sequencing (SLAF-seq) of F2 population | 260380 | Yi et al., 2017 |
| SNPs | *Linum usitatissimum* | Genotyping by sequencing (GBS) of 370 accessions from the flax core collection | 258,873 | He et al., 2018 |
| SNPs | *Linum usitatissimum* | Specific locus amplified fragment sequencing (SLAF-seq) of 224 varieties | 584,987 | Xie et al., 2018a |
| SNPs | *Linum usitatissimum* | Genome sequencing of flax core- 383 accessions | 51,575 | Sertse et al., 2019 |
| SNPs | *Linum usitatissimum* | Genome resequencing of 200 flax cultivated accessions | 1,179,402 | Guo et al., 2020 |
| SNPs | *Linum usitatissimum* | Genotyping by sequencing (GBS) of 131 accessions | 68,925 | Saroha et al., 2022b |

**Supplementary Table 3: Genome Wide Association Studies (GWAS) undertaken in linseed**

| **Trait** | **QTLs/**  **QTNs/ Associated SNP loci** | **Accessions in AM panel** | **Phenotyping environments** | **Genotyping method and markers used** | **Method used for GWAS** | **Reference** |
| --- | --- | --- | --- | --- | --- | --- |
| Pasmo resistance | 500 | 370 | Five years | GBS, 258,873 SNPs | 10 Single- and multi-locus methods | He et al., 2018 |
| Agronomic & fiber traits, Fatty acid content | 23 | 224 | Single year with three replicates, lab-based analysis | SLAF-seq, 584,987 SNPs | Efficient mixed model (EMMAX) and GLM | Xie et al., 2018a |
| Agronomic traits | 42 | 224 | Three environments | SLAF-seq, 584,987 SNPs | GLM, MLM | Xie et al., 2018b |
| Agronomic traits | 12 | 390 | Eight environments | SSRs, 464 | Mixed model (PCA + kinship matrix (K) | Soto-Cerda et al., 2014a |
| Seed quality traits | 9 | 390 | Six environments | SSRs, 460 | GLM | Soto-Cerda et al., 2014b |
| Mucilage and hull Content | 11 | 200 | Two environments | GBS, 1.7 million SNPs | GLM-Q, GLM-PCA, and MLM-K | Soto-Cerda et al., 2018 |
| Seed yield and oil quality | 33 | 260 | Six-Eight environments | GBS, 17,288 SNPs | GLM and MLM | You et al., 2018 |
| Early root and shoot traits | 228 | 115 | Three biological replicates under controlled conditions | Resequencing, 7000 & 3000  SNPs | GLM, MLM, FASTmrEMM, FASTmrMLM, ISIS EM-BLASSO, mrMLM, pKWmEB &  pLARmEB; LFMM | Sertse et al., 2019 |
| Seed size and weight | 599 | 200 | Four environments | Resequencing, 674,074  SNPs | MLM | Guo et al., 2020 |
| Flowering time | 40 | 200 | Four environments | Resequencing, 70,935 SNPs | GLM, MLM, FASTmrEMM, FASTmrMLM, ISIS EM-BLASSO, mrMLM, pKWmEB &  pLARmEB | Soto-Cerda et al., 2021 |
| Flowering time, maturity & plant height | 109 | 131 | Five environments | GBS, 68,925 SNPs | FASTmrEMM, FASTmrMLM, ISIS EM-BLASSO, mrMLM, &  pLARmEB | Saroha et al., 2022b |
| Seed yield, days to maturity, iodine value, protein, oil, linoleic acid, & linolenic acid content | 1420 | 260 | Four years over two locations | GBS 17,277 SNPs | FASTmrEMM, FASTmrMLM, ISIS EM-BLASSO, mrMLM, pKWmEB &  pLARmEB; GLM, MLM, RTM-GWAS | Lan et al., 2020 |
| Powdery mildew resistance | 349 | 447 | Four years over two locations | 247,160 SNPs | GLM, MLM, pLARmEB, pKWmEB, FASTmrMLM, ISIS EM-BLASSO, mrMLM, FASTmrEMMA, and FarmCPU | You et al., 2022 |
| Salt tolerance at germination | 64 | 200 | Three replications under controlled conditions | Resequencing, 674,074  SNPs | GLM, MLM | Li et al., 2022 |
